# Untargeted Metabolomics Coupled with Chemometrics for Leaves and Stem Barks of Dioecious *Morus alba* L.

**DOI:** 10.3390/metabo12020106

**Published:** 2022-01-24

**Authors:** Cui Wu, Huijun Wang, Zhenying Liu, Bo Xu, Zhuojun Li, Pingping Song, Zhimao Chao

**Affiliations:** Institute of Chinese Materia Medica, China Academy of Chinese Medical Sciences, Beijing 100700, China; wucuidalian@163.com (C.W.); hjwang0819@icmm.ac.cn (H.W.); liuzy9607@163.com (Z.L.); xubo_345@163.com (B.X.); 18811385399@163.com (Z.L.); songpingping122@163.com (P.S.)

**Keywords:** *Morus alba* L., dioecious plant, leaves, stem barks, untargeted metabolomics, chemometrics

## Abstract

The differences in metabolites in male and female individuals of dioecious *Morus alba* L. (Moraceae) are usually ignored and lack study. In the present study, 58 leaves and 61 stem barks from male and female individuals were analyzed by untargeted metabolomics via headspace solid-phase microextraction gas chromatography-mass spectrometry (HS-SPME-GC-MS) coupled with chemometrics, including principal component analysis (PCA) and orthogonal partial least squares-discriminant analysis (OPLS-DA). A total of 66 and 44 metabolites were identified from leaves and stem barks, respectively. Four and eight differential metabolites among candidate metabolites in leaves and stem barks from male and female individuals were identified. Moreover, females possessed stronger antioxidant activity than males. This is the first report where untargeted metabolomics coupled with chemometrics was used to analyze the different metabolites and to discriminate the gender of leaves and stem barks of dioecious *M. alba*. It provided the basis for further study of *M. alba* and reference value for researching dioecious plants.

## 1. Introduction

*Morus alba* L., a typical dioecious plant, is native to China and has been widely cultivated and naturalized elsewhere, such as American, Japan, and Korea. For thousands of years, various parts of *M. alba* have been used in traditional Chinese medicine (TCM), food, agriculture, and other areas. Leaves are edible and can be used to make tea. In addition, leaves can be used as functional nutraceutical food in people’s daily lives to preserve health [1], as forage for silkworms in sericulture, and as a herb in TCM to treat several diseases especially diabetes mellitus type II [2,3]. As the largest part of *M. alba*, researches on leaves mainly focus on the chemical components [4], species [5], and pharmacological activities [6]. Twigs and root barks are traditional herb medicines for treating arthritis and cough in the clinic [2]. Fruits are also used in TCM to protect against liver and kidney damage, strengthen the joints, improve eyesight, and in food to make hair black and improve human health [7,8]. Stem barks are considered the origin of fiber and vermifuge and purgative in the Indian traditional system of medicine [9]. Attention was paid to the component’s isolation and pharmacological activities of stem barks [10,11].

Dioecious plants refer to species having distinct male and female individuals. Large differences exist in biological features between the male and female individuals of dioecious plants, such as physiological, biochemical, and ecological adaptability [12,13,14]. Recently, research have been carried out on the chemical differences and discrimination of male and female individuals of dioecious plants such as barks and flower buds of *Populus tomentosa* with high-performance liquid chromatography (HPLC), ^1^H-nuclear magnetic resonance (^1^H-NMR), and headspace solid-phase microextraction gas chromatography-mass spectrometry (HS-SPME-GC-MS) in combination with chemometrics [14,15,16]. For *M. alba*, research on male and female individuals of *M. alba* focused on sex determination mechanisms and effects of the environment on mulberry saplings’ growth [17,18]. There are no research on comparing the metabolites of male and female individuals and discriminating their gender.

The untargeted metabolomics has the advantage of comparing multiple complex products without any a priori knowledge of their composition and identification of major metabolites [19]. It has been a steady growth due to the development and implementation of affordable and high-quality metabolomics platforms. The chemometrics can extract significant information from the raw data and reveal latent correlations existing between a multitude of variables [20]. Therefore, untargeted metabolomics combined with chemometrics has become an important and valuable tool in various research of life science such as biomarker discovery, disease diagnosis, and quality evaluation of food and herb recently. Ultra-performance liquid chromatography-tandem quadrupole time-of-flight mass spectrometry (UPLC-Q-TOF-MS) is one of the most frequently used techniques for untargeted metabolomics depending on its high separation capacity, sensitivity, and accuracy [21]. In a quadrupole time-of-flight mass spectrometry (Q-TOF-MS) analysis, each precursor ion selected by the quadrupole mass analyzer is dissociated in the collision cell, and the generated fragment ions are then detected successively by the TOF analyzer, which allows it to detect a wide range of chemicals effectively in a few minutes. In addition, it can provide accurate mass measurement and sufficient fragment information for metabolite identification [22].

Therefore, in the present study, 58 leaf samples and 61 stem bark samples from both male and female individuals of *M. alba* were collected. The analysis of metabolites and the gender discrimination of male and female leaves and stem barks were carried out by untargeted metabolomics based on UPLC-Q-TOF-MS combined with chemometrics, including principal component analysis (PCA) and orthogonal partial least squares-discriminant analysis (OPLS-DA). Moreover, the antioxidant activity of males and females was evaluated.

## 2. Results and Discussion

### 2.1. Untargeted Analysis of Samples

The raw data pre-processed with Progenesis QI gave evaluated parameters including RT, score, mass error (ME), fragmentation score (FS), and isotopic similarity (IS). Then metabolites were confirmed by the following ways: (1) comparing RT, MS spectra, and MS/MS spectra with authentic standards; (2) comparing RT, MS spectra, and MS/MS spectra with published literature; (3) comparing RT, score, ME, FS, and IS with an in-house library or web-accessible database. As a result, a total of 66 metabolites were definitely or tentatively identified from leaf samples by chemical standards or the established in-house and web-accessible database, including 27 flavonoids, 9 phenolic acids, 6 organic acids, 5 coumarins, 4 stilbenes, 3 benzofurans, 2 amino acids, 2 alkaloids, 2 saccharides, and others (Table S1). The representative total ion current chromatogram (TIC) for leaf sample (LF21) was shown in Figure S1A,B. The main components of leaf samples were flavonoids, which possessed activities such as antibacterial [23], antioxidant [24], hepatoprotective and neuroprotective [25], and α-glucosidase inhibitory [26]. In the present study, flavonoids could be divided into three types: flavonol, flavone, and flavanone and their glycosides. Flavonol, including 18 metabolites, were present as glycoside derivatives, e.g., quercetin and kaempferol attached to sugars such as glucose, rhamnose, and malonylglucose. Flavone and its glycosides mainly consisted of luteolin, apigenin, dehydrocyclomorusin, luteolin-7-diglucoside, and chrysin. Flavanone mainly were naringenin, taxifolin, euchrenone a7, and plantagoside. Organic acids exist widely and play versatile stress response roles in plants [27]. Plants’ phenolic acids are considered the functional food ingredients and potential therapeutic components [28,29]. Coumarins have been paid considerable attention in recent years with significant soluble epoxide hydrolase inhibitory, anti-inflammatory, antipyretic, and analgesic activities [30,31].

A typical TIC of stem bark sample of *M. alba* (BF21) was shown in Figure S1C. In the untargeted metabolomics, a total of 44 metabolites were identified, which were divided into 10 categories, including 10 flavonoids, 7 organic acids, 6 benzofurans, 6 stilbenes, 5 alkaloids, 2 amino acids, 2 phenolic acids, 2 iridoids, and others (Table S2). Flavonoids in the stem bark of *M. alba* mainly consisted of flavanones (kuwanon G, cyclocommunol, 10-oxomornigrol F, and mornigrol F), flavonol glycosides (Kaempferol di-O-hexoside and hyperoside), flavanone glycosides (plantagoside and prunin), flavanonol glycoside (astilbin), and dihydrochalcone glycoside (phloridzin). Flavonoids have attracted attention because of their cardioprotective [32], antiviral [33], hepatoprotective, and neuroprotective activities [25]. Benzofurans have anticancer, antimicrobial, immunomodulatory, antioxidant, and anti-inflammatory activities [34]. Stilbenes have antitussive and anti-asthmatic functions [35].

The metabolites in leaves and stem barks of *M. alba* were analyzed by untargeted metabolomics for the first time. The metabolic profiling of leaves provides a basic foundation for further study. The untargeted metabolomics of stem barks fills the gap in the study of metabolites for stem barks. Flavonoids were the main metabolites of leaves and stem barks, which play a wide range of physiological and ecological roles in plants [36]. Leaves are the terminal part of the plant. Stem barks are responsible for storing and transporting nutrients. The identification of their metabolites lays a foundation for further researches.

### 2.2. The Metabolome of Male and Female Leaves

**Figure 1:**
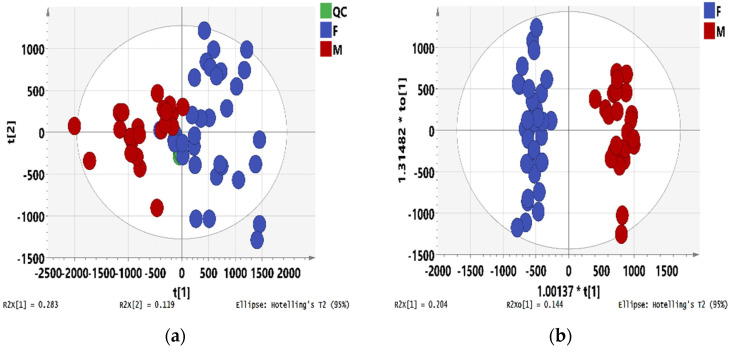

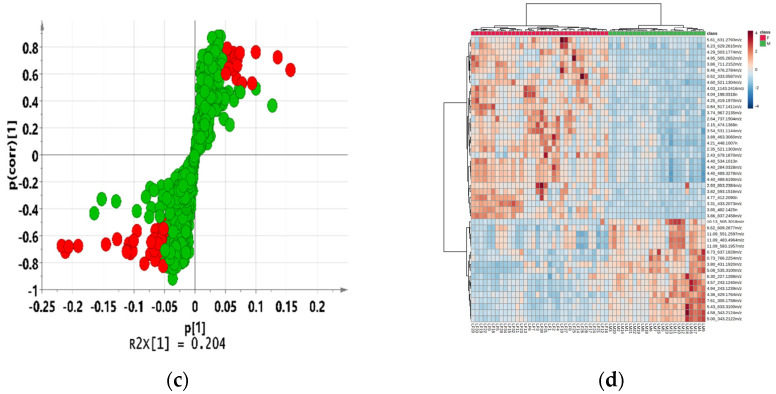
Chemometrics of male and female leaf samples. (**a**) PCA score plot; (**b**) OPLS-DA score plot; (**c**) S-plot of OPLS-DA along with selected candidate differential metabolites (cut-off values of |p| ≥ 0.05 and |p(corr)| ≥ 0.5); (**d**) Heat-map and hierarchical clustering analysis (HCA) analyses of metabolite contents in male and female leaf samples.

UPLC-ESI-Q-TOF-MS analyzed both male and female leaf samples. These data were pre-processed by Progenesis QI and then imported to SIMCA-P for PCA and OPLS-DA analysis. The scale type was set to Pareto scaling mode. QC sample was employed to validate the chromatographic and mass detection system to investigate the data reliability. In the PCA score plot (Figure 1a), the first principle accounted for 28.3%, while the second principle accounted for 11.9% of the total variance. The QC samples clustered into one small set near the coordinate origin, indicating that the stability and reproducibility of the experiment could be guaranteed.

The score plot of OPLS-DA shows a clear separation of male and female leaf samples (Figure 1b). To statistically validate the model, the default 7-fold cross-validation, CV-ANOVA analysis, and permutation test (200 permutations) were performed [37,38,39]. The R^2^ of the OPLS-DA model was 0.964 indicating the model was well fitted. The Q^2^ was 0.908, indicating a good predictivity. CV-ANOVA analysis (*p* < 0.001) demonstrated that the obtained OPLS-DA models were highly significant. The results of permutation tests (200 permutations) showed that the intercept value of Q^2^ was lower than 0.05 (Figure S2A). These validations showed that the model was statistically valid and that the high value of predictability did not arise from overfitting. As shown in Figure 1c, variables far from coordinate mean more contribution to the differentiation of groups. Values of |*p*| ≥ 0.05 and |*p*(corr)| ≥ 0.5 in S-plot and VIP > 1 were used to screening differential metabolites responsible for the differentiation. Subsequently, these tagged markers were filtered with ANOVA *p* value ≤0.05 and max fold change ≥1.5. As a result, 47 candidate retention time-exact mass (RT-EM) pairs were selected and used to plot an HCA heatmap to find relatively homogeneous clusters of samples and to illustrate the content changes of metabolites in different groups of samples. As shown in Figure 1d, 24 male and 34 female leaf samples were divided into two groups under HCA. Four of 47 candidate RT-EM pairs were identified by comparing the retention behavior, accurate molecular weight, and MS^E^ fragments with those in the literature (Table 1). Compound L1 yielded the parent ion at *m*/*z* 447.1328 [M−H]^−^ and fragment ions at *m*/*z* 285.0392 and 151.0037 were observed in the MS/MS spectrum. Compound L1 was identified as kaempferol 3-O-glucoside (astragalin), reported in the literature [40]. For compound L2, a parent ion at *m*/*z* 533.0931 [M−H]^−^ and fragment ions at *m*/*z* 285.0432 and 151.0036 matched the mass data of kaempferol 3-O-(6″-O-malonylglucoside) [4]. For compound L3, the parent ion at *m*/*z* 737.1904 [M−H_2_O−H]^−^ and fragment ion at *m*/*z* 301.0350 were observed. After further comparison with the literature, compound L3 was identified as quercetin O-rhamnosyl-O-rhamnosyl-O-hexoside [41]. Compound L4 displayed ions of *m*/*z* 593.1516 [M−H]^−^, 285.0381, and 151.0034 in negative mode, which was identified as kaempferol O-rhamnosyl-O-hexoside by comparing with reported literature [42]. Astragalin (L1) is a flavonol glycoside found in a variety of plants with many biological functions such as attenuating allergic inflammation [43], inducing selective kidney cancer cell death [44], and inhibiting autophagy-associated airway epithelial fibrosis [45]. Kaempferol 3-O-(6″-O-malonylglucoside) (L2) is a bioactive component in leaves of *M. alba* with antidiabetic and antioxidant activities [46,47]. The literature from Dong Li et al. showed that these differential metabolites were involved in flavonoid biosynthesis [4].

As shown in Figure 1d, the contents of 33 RT-EM pairs in male leaf samples were lower than those in females, and the contents of other 14 RT-EM pairs in male leaf samples were higher than those in females. These inequalities indicated that the efficacy of leaves from male and female individuals might be different, which had an important reference for the development and utilization of leaves. This is the first report on the different metabolites of male and female leaves.

Leaves are the vegetative organ of *M. alba*. Gender can not be judged morphologically. Untargeted metabolomics combined with chemometrics can well discriminate the male and female leaves, which provided reference value for other dioecious plants. The discovery of differences in their metabolites in males and females will provide targeted direction to develop functional food products and special medical products related to gender.

### 2.3. The Metabolome of Male and Female Stem Barks

**Figure 2:**
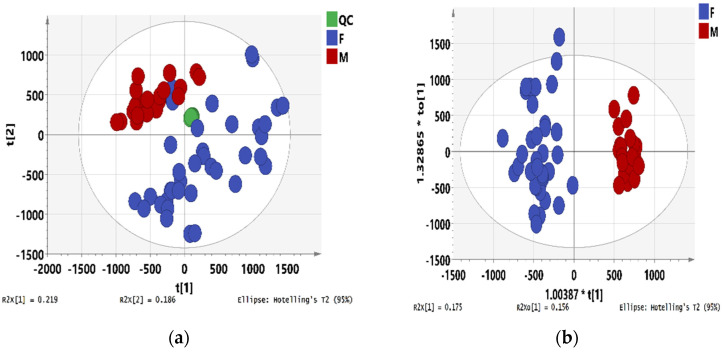

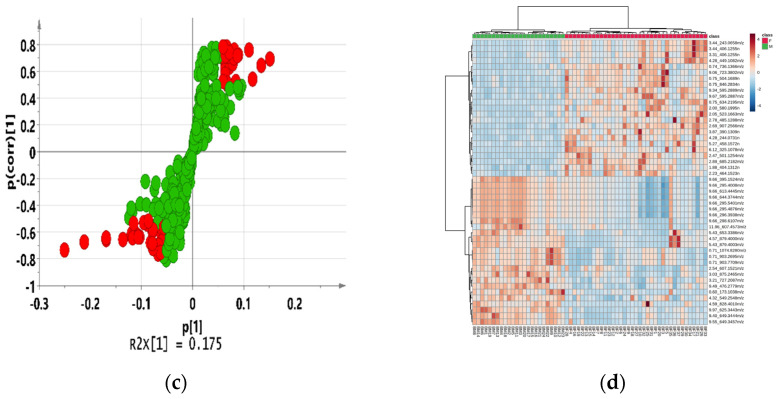
Chemometrics of male and female stem bark samples. (**a**) PCA score plot; (**b**) OPLS-DA score plot; (**c**) S-plot of OPLS-DA along with selected candidate differential metabolites (cut-off values of |p| ≥0.05 and |p(corr)| ≥ 0.5); (**d**) Heat-map and HCA analyses of metabolite contents in male and female stem bark samples.

Both 24 male and 37 female stem bark samples of mature *M. alba* were analyzed with UPLC-ESI-Q-TOF-MS combined with chemometrics to find chemical differences and discriminate gender. As shown in the score plot of the PCA model (Figure 2a), the first and the second principal components explained 21.9% and 18.6% of the variation, respectively. The position of QC samples was near the coordinate origin. In the score plot of OPLS-DA, male and female stem bark samples were clustered into two groups, indicating significant differences in the chemical compositions of these two groups (Figure 2b). The R^2^ and Q^2^ were 0.935 and 0.855, respectively, which indicated a good degree of fit and predictive ability of the established model. Moreover, CV-ANOVA analysis (*p* < 0.001) and intercept value of Q^2^ from permutation test (Figure S2B) showed that the model was statistically valid.

In S-plot (Figure 2c), values of |*p*| ≥ 0.05 and |*p*(corr)| ≥ 0.5 were chosen in combination with values of VIP > 1, ANOVA *p* value ≤ 0.05, and max fold change ≥1.5 for screening differential metabolites. An HCA heatmap was plotted and shown in Figure 2d, in which male and female stem barks were clustered into two groups. Finally, 48 RT-EM pairs were discovered from the comparative analysis and considered potentially differential metabolites of male and female stem barks. Eight RT-EM pairs of them were identified and shown in Table 2.

Compound B1 yielded the parent ion at *m*/*z* 173.1038 [M−H]^−^ and was identified as arginine according to the literature [48]. Compound B2 displayed the parent ion at *m*/*z* 503.1606 [M−H]^−^ and fragment ions at *m*/*z* 221.0655, 179.0552, 161.0447, 113.0236, 89.0240, and 71.0135 and was identified as raffinose by comparing with those ions recorded in Massbank. For compound B3, the parent ion at *m*/*z* 403.1235 [M−H]^−^ and fragment ions at *m*/*z* 191.0556 and 119.0315 were observed. After further comparison with the literature [49], compound B3 was identified as gardenoside. Compound B4 showed the parent ion at *m*/*z* 405.1161 [M−H]^−^ and fragment ion at *m*/*z* 243.0649. It was identified as astringin by comparing the retention behavior, accurate molecular weight, and MS^E^ fragments with the literature [50]. Compound B5 yielded the parent ion at *m*/*z* 389.1222 [M−H]^−^ and fragment ion at *m*/*z* 227.0703 was observed in the MS/MS spectrum, corresponding to the mass data of piceid [51]. Compound B6 exhibited an abundant parent ion at *m*/*z* 243.0653 [M−H]^−^ and fragment ions at *m*/*z* 225.0549, 199.0755, and 175.0756, and was identified as oxyresveratrol by comparing with a standard. Compound B7 and B8 were identified as mulberroside C and moracinfurol A by confirming with ME, FS, IS, and the score matched with an in-house library, respectively. Arginine (B1) is an important amino acid that is not only required for protein synthesis but is also an intermediate for nitrogen storage and a precursor of compounds that act as second messengers in developmental processes, such as polyamines and nitric oxide [52,53,54,55]. It plays a major metabolic role in seed maturation, germination, phloem and xylem transport, and accumulates under stress conditions [56]. Therefore, as the differential metabolites in male and female stem barks, arginine may induce a series of metabolic differences and finally result in different metabolites. Raffinose (B2) serves as a desiccation protectant in seeds, transporting sugar in phloem sap and storing sugar in plants [57]. Gardenoside (B3) possesses antidepressive activity [58] and pain suppression [59]. Astringin (B4) is a natural glycoside found in the stem barks of *M. alba*, *Picea sitchensis*, and *P. abies* (Norway spruce, Family Pinaceae) and has antioxidant, potential cancer-chemopreventive, and antimicrobial activities [60,61,62]. Piceid (B5), a naturally occurring glucoside of resveratrol found in many plants, has recently been considered a potential nutraceutical [63]. Mulberroside C (B7) is a 2-arylbenzofuran derivative in the root barks of *M. alba* with antiviral and antiplatelet activities [64,65,66].

Oxyresveratrol (B6), resveratrol with one more hydroxyl group, is abundant in the wood of *M. alba* and has biological effects, including against tyrosinase and prevention of acute liver injury induced by lipopolysaccharide/D-galactosamine [67,68]. The results of the semi-quantitation of oxyresveratrol were calculated as shown in Table S3. The content of oxyresveratrol in female stem bark samples varied from 2.085 to 24.18 μg/g with an average content of 11.26 μg/g while varied from 0.766 to 8.481 μg/g in male samples with an average content of 3.051 μg/g. These results indicated that the female stem barks contained more oxyresveratrol than males. As shown in the heatmap (Figure 2d), the contents of 23 RT-EM pairs in male stem barks were lower than those in females, and the contents of other 25 RT-EM pairs in female stem barks were lower than those in males.

**Figure 3:**
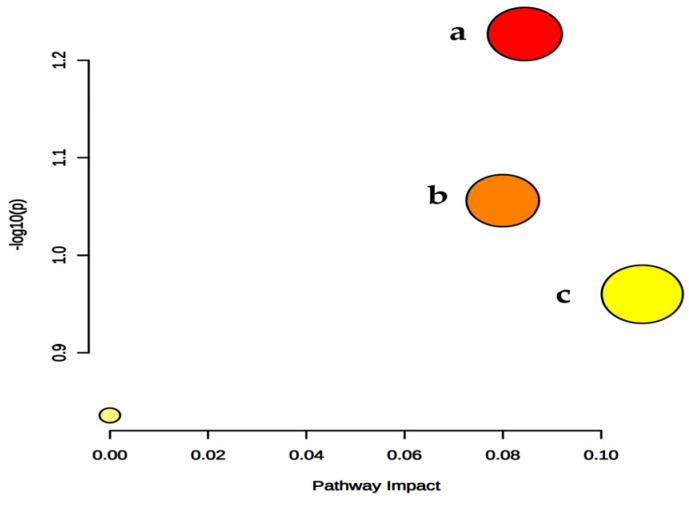
The bubble chart of metabolic pathway analysis. **a** Arginine biosynthesis; **b** Galactose metabolism; **c** Arginine and proline metabolism.

For metabolic pathway analysis, these differential metabolites were imported into metaboanalyst (http://www.metaboanalyst.ca/, accessed on 17 December 2022). As shown in Figure 3, the node with a high impact value suggested potential targeted pathways, arginine biosynthesis, galactose metabolism, and arginine and proline metabolism were identified as important targeted pathways with high impact values. The metabolic route to arginine synthesis in plants and other organisms involves two distinct processes: synthesis of L-ornithine (Orn) from glutamate and arginine synthesis from the Orn intermediate [69]. Raffinose is consistent with galactose, fructose, and glucose. The galactose metabolism is relevant to raffinose [70]. Regulating these targeted pathways may achieve purposeful intervention in the expression of metabolites. At present, there are few studies on these metabolic pathways in *M. alba*. The results can also provide a reference for relevant studies of targeted pathways.

At present, the comparison of male and female stem barks of *M. alba* is still blank. The results of this experiment have important reference value for the further research, development, and utilization of stem barks of *M. alba*.

### 2.4. The Comparison of Antioxidant Activity of Males and Females

The antioxidant activity, a nonspecific evaluation, is widely carried out in natural products. Due to the complex composition of the phytochemical and oxidative processes, the antioxidant activity of plant extracts cannot be evaluated using only one method [71]. Therefore, at least two methods should be employed to evaluate the total antioxidant activity. In the present study, 2,2-diphenyl-1-picrylhydrazyl (DPPH) and 2,2′-azinobis (3-ethylbenzo thiazoline-6-sulfonic acid) diammonium salt (ABTS) were employed to evaluate the antioxidant activity of male and female leaves and stem barks of *M. alba*. The results are shown in Tables S4 and S5. The student’s *t*-test was performed on the SPSS 20.0 software to analyze the significance of the values between males and females. For both leaves and stem barks, the DPPH and ABTS values of females were significantly higher than those in males, which demonstrated that the antioxidant activities of females were stronger (Tables S6 and S7). It is suggested that there may be differences in the pharmacological activities of male and female individuals of dioecious *M. alba*, which is worthy of further study to develop and use them purposefully.

## 3. Materials and Methods

### 3.1. Plant Materials

In June 2021, 58 leaf samples (24 males and 34 females) and 61 stem bark samples (24 males and 37 females) were harvested from the planting base of *M. alba,* which was located at 39°48′50.97″ north latitude, 116°02′48.59″ east longitude, and 108 m altitude in Shangwan Village, Qinglonghu Town, Fangshan District, Beijing. The male individuals were identified based on the gardener’s records of previous years for their male flowers, and female individuals were identified based on their fruits. All samples were collected from the mature *M. alba* with 40–55 cm girth. The leaf samples were picked from these trees with complete, pollution-free, and multi-directional states. The stem barks with a width of 5 cm and the height of 15 cm were stripped off at the height of 0.6–0.7 m of the trunk. The leaves and stem barks were dried under outdoor sunlight for 3 and 5 days, respectively. The phellem of the dried stem barks was scraped off, and the rest phloem was used as the experimental samples. The botanical origin of the samples was identified by Prof. Zhimao Chao (Institute of Chinese Materia Medica, China Academy of Chinese Medical Sciences) according to the description in Flora of China and the voucher specimens (BF1−37, BM1−24, LF1−34, and LM1−24. B: stem bark, L: leave, F: female, and M: male) were deposited at the 1022 room of Institute of Chinese Materia Medica, China Academy of Chinese Medical Sciences.

### 3.2. Chemicals and Standards

Methanol, acetonitrile, and formic acid of HPLC grade were purchased from ThermoFisher Scientific (Shanghai, China). Ultrapure water was produced using a Milli-Q water purification system (Millipore, Billerica, MA, USA). Authentic standards of apigenin, chlorogenic acid, kuwanon G, luteolin, mulberroside A, naringenin, and oxyresveratrol were purchased from Push-Biotechnology Co., Ltd. (Chengdu, China). DPPH and ABTS were acquired from Shanghai Aladdin Bio-Chem Technology Co., Ltd. (Shanghai, China). 6-Hydroxy-2,5,7,8-tetramethylchromane-2-carboxylic acid (Trolox) was obtained from Sigma-Aldrich (St. Louis, MO, USA). All other solvents and reagents were of analytical grade.

### 3.3. Preparation of Sample and Standards Solution

Seven standards of apigenin, chlorogenic acid, kuwanon G, luteolin, mulberroside A, naringenin, and oxyresveratrol were weighed accurately and dissolved in methanol to prepare a mixed-standard stock solution. The final concentrations for the standard solution were 8.68, 8.48, 8.08, 8.60, 8.72, 7.60, and 8.24 μg/mL in methanol, respectively.

The dried leaf and stem bark samples were crushed into a powder with a grinder and passed through 50-mesh and 24-mesh sieves, respectively. The sample powders (0.5 g each) were weighed accurately in a 50-mL stoppered conical flask, and 25 mL of 50% methanol was added. The solutions were weighed and extracted by an ultrasonator (100 W and 40 kHz, KQ-100E, Kunshan Ultrasonic Instruments Co. Ltd. Kunshan, China) for 45 min, and then were weighed again after cooling. Weight losses from sonication of the extract were replaced with 50% methanol. The obtained solutions were filtered through a 0.22 μm of polytetrafluoroethylene (PTFE) filter membrane.

Two quality control (QC) sample solutions, both leaf and stem bark samples, were prepared by mixing an equal amount of each leaf and stem bark sample in 50% methanol, respectively. These QC samples served as technical replicates to supervise the repeatability and stability of the analytical system and were analyzed every 10 injections. The 50% methanol used in the sample preparation served as the blank control to verify the cleanliness of the UPLC-Q-TOF-MS system. The blank response was subtracted as the background correction in the data processing.

### 3.4. UPLC-Q-TOF-MS System and Analytical Conditions

Metabolomics analysis was performed on a UPLC-Q-TOF-MS system consisting of a Waters ACQUITY I-class UPLC and Xevo G2-XS Q-TOF mass spectrometer (Waters Corporation, Milford, MA, USA), equipped with an electrospray ionization (ESI) source (Waters Corporation, Milford, MA, USA). Chromatographic separation was achieved using an ACQUITY UPLC HSS T3 (2.1 mm × 100 mm, 1.8 μm, Waters). The mobile phase consisted of solvent A (H_2_O containing 0.1% formic acid, *v*/*v*) and solvent B (acetonitrile containing 0.1% formic acid, *v*/*v*). The gradient elution was performed as following protocol: 0–12 min, 2–95% B; 12–13 min, 95% B; 13–13.01 min, 95–2% B; and 13.01–15 min, 2% B. The column was kept at 45 °C, and the flow rate was 0.4 mL/min. The injection volume was 1.0 μL.

The MS was operated in negative ionization mode across a scan range of *m*/*z* 50 to 1200 with a scan time of 0.25 s. Source parameters were as follows: capillary voltage, 2.5 kV; sampling cone voltage, 40 V; source temperature, 120 °C; cone gas, 50 L/h; desolvation temperature, 450 °C; and desolvation gas flow, 800 L/h. Argon (99.95%) was used for collision-induced dissociation, and N_2_ was used as the drift gas. The low collision energy was set to 6 eV, and the high collision energy was ramped from 15 to 50 eV. MS^E^ analysis was employed to simultaneously acquire the exact mass of small molecules at high and low collision energy. The MS was calibrated daily by Waters calibration standard. Leucine enkephalin was used for lock mass correction with *m*/*z* 554.2615.

### 3.5. Data Processing

The raw data obtained from UPLC-Q-TOF-MS were collected using the MassLynx4.1 (Waters Corporation, Milford, MA, USA) and then imported to the Progenesis QI software (Nonlinear Dynamics, Newcastle, UK). Data were processed in successive treatment steps as normalization, peak alignment, peak picking, experiment design setup, deconvolution, and metabolites identification, and a data matrix was obtained. Samples of LF21 and BF21 were selected randomly as a reference of leave, and stem bark samples for peak alignment and the blank response were subtracted, respectively. The peak picking parameters were set as follows: all runs, absolute ion intensity: 5000, retention time (RT): 0–12 min, adduct ion forms: [M−H]^−^, [M+FA−H]^−^, [M−H_2_O−H]^−^, or [2M−H]^−^. Subsequently, the algorithm of ANOVA *p* value and max fold change was used to filter RT-EM pairs.

### 3.6. Chemometrics

The raw data were imported to Progenesis QI for pretreating, including normalization, peak alignment, and peak picking. The resultant datasets of leaf and stem bark samples, comprising the sample code, *m*/*z*, peak RT, and peak areas, were imported into the SIMCA-P software package (Version 14.1 Umetrics, Umea, Sweden) to conduct chemometrics, including principal component analysis (PCA) and orthogonal partial least squares-discriminant analysis (OPLS-DA). PCA, an unsupervised pattern recognition pattern, reduces the dimension of the data matrix and converts the original variables into a new independent variable called principal components (PCs). It reveals the interrelationships between different variables and interprets sample patterns, groupings, similarities, and differences. OPLS-DA uses the class membership to maximize the variation, introduce an orthogonal signal correction (OSC) filter to separately handle the systematic variation correlated to or uncorrelated to the Y variable, and therefore, have the better discriminant ability for the samples with larger within-class divergence than PCA. The quality and reliability of these models are usually evaluated with R^2^ and Q^2^. Their values ranged from 0 to 1, where 1 indicates perfect fitness and predictivity of models. Variable importance for projection (VIP) values generated from the OPLS-DA model indicates the most important variables to classification. In the S-plot of OPLS-DA, covariance (p) corresponds to the contribution of the ions to the variance of the observations. Correlation p(corr) represents the correlation between samples and the reliability of the results. In addition, ANOVA *p* value and max fold change were employed to identify the differential metabolites that contributed to the differentiation of groups. Finally, RT-EM pairs filtered with the abovementioned parameters were employed for hierarchical clustering analysis (HCA) heatmap using Metaboanalyst to visualize the differences between groups intuitively.

### 3.7. Metabolites Identification and Semi-Quantitation Analysis

An in-house library that could rapidly search the known metabolites from complex mass spectra data was established. First, the metabolites name of *M. alba* from the literature were collected, and the structure files (.mol or .sdf) of each metabolite were downloaded from PubChem, chemsprider, chemicalbook, or manually drawn by ChemDraw. Then all structure files were combined as a Structure Data File (SDF) by Progenesis SDF studio. Finally, the Progenesis Metascope search of the SDF database was carried out assuming 10 ppm error for precursor ions and 10 ppm for theoretical fragments.

In addition, metabolites were also identified using the Progenesis QI automatic based on the accurate mass, MS/MS fragments, and isotope label by searching HMDB (http://www.hmdb.ca/, accessed on 21 October 2021), KEGG (https://www.kegg.jp/, 21 October 2021), Pubmed (https://pubmed.ncbi.nlm.nih.gov/, accessed on 21 October 2021), and Massbank (http://www.massbank.jp/, accessed on 21 October 2021) public databases.

For differential metabolites identified by standard, the semi-quantitation was calculated by comparing the peak areas of standard and normalized peak areas of samples.

### 3.8. Determination of Antioxidant Activity

The Trolox equivalent antioxidant capacity of leaves and stem barks of *M. alba* was analyzed using the DPPH and ABTS assays with a TU-1810ASPC ultraviolet, visible spectrophotometer (Purkinje General Instrument Co., Ltd., Beijing, China).

The DPPH assay was determined following the method reported with slight modifications [72]. Briefly, 200 μL aliquot of each sample was mixed with 0.2 mM of DPPH in methanol and brought to a final volume of 5.0 mL. After 30 min of incubation in the dark, the absorbance at 517 nm was measured. The ABTS assay was conducted as previously described [73] with slight modifications. A dark-blue-colored stock solution was prepared by dissolving 7 mM ABTS in a 140 mM potassium persulfate solution, storing this solution in the dark for 16 h at room temperature. The solution was then diluted to reach an absorbance of 0.7 ± 0.02 at 734 nm. Then 20 μL of each sample was added to the 3.0 mL ABTS solution. After 5 min in the dark, absorbance was measured at 734 nm. The Trolox solutions were used to obtain the calibration curves. The results are expressed as mg equivalents of Trolox per gram of sample (mg TE/g sample). All experiments were performed in triplicate.

## 4. Conclusions

In this study, we investigated the metabolites of leaves and stem barks of dioecious *M. alba* by untargeted metabolomics, analyzed the differential metabolites of males and females combined with chemometrics, and evaluated the antioxidant activity of males and females.

A total of 66 and 44 metabolites were identified in leaves and stem barks, respectively. Kaempferol 3-O-glucoside, kaempferol 3-O-(6″-O-malonylglucoside), quercetin O-rhamnosyl-O-rhamnosyl-O-hexoside, and kaempferol O-rhamnosyl-O-hexoside were the differential metabolites in male and female leaves. Arginine, raffinose, gardenoside, astringin, piceid, oxyresveratrol, mulberroside C, and moracinfurol A were the differential metabolites in male and female stem barks. Moreover, the antioxidant activity of females was stronger than males. This study lays a foundation for further research, development, and utilization for dioecious *M. alba.* Many dioecious plants are used in food, medicine, agriculture, and other fields, with few studies on the identification and comparison of male and female individuals. Therefore, this study can also provide a valuable reference for studying dioecious plants.

## Figures and Tables

**Table 1:** Differential metabolites for male and female leaves, filtered with VIP > 1, |*p*| ≥ 0.05 and |*p*(corr)| ≥ 0.5, ANOVA *p* value ≤ 0.05, and max fold change ≥ 1.5.

No.	RT-EM	Mass Accuracy (ppm)	Formula	VIP	Max Fold Change	Compound	Reference
L1	4.21_448.1007n	0.25	C_21_H_20_O_11_	5.74	1.61	Kaempferol 3-O-glucoside (astragalin)	[40]
L2	4.40_534.1013n	0.72	C_24_H_22_O_14_	5.67	1.60	Kaempferol 3-O-(6’’-O-malonylglucoside)	[4]
L3	2.64_737.1904*m*/*z*	−4.06	C_33_H_40_O_20_	1.33	1.72	Quercetin O-rhamnosyl-O-rhamnosyl-O-hexoside	[41]
L4	3.82_593.1516*m*/*z*	0.70	C_27_H_30_O_15_	3.61	1.74	Kaempferol O-rhamnosyl-O-hexoside	[42]

n neutral molecular weight calculated according to adduct ion forms.

**Table 2:** Differential metabolites for male and female stem barks, filtered with VIP > 1, |*p*| ≥ 0.05 and |*p*(corr)| ≥ 0.5, ANOVA *p* value ≤ 0.05, and max fold change ≥ 1.5.

No.	RT-EM	Mass Accuracy (ppm)	Formula	VIP	Max Fold Change	Compound	Reference
B1	0.60_173.1038*m*/*z*	−3.47	C_6_H_14_N_4_O_2_	1.03	3.69	Arginine	[48]
B2	0.75_504.1689n	−0.23	C_18_H_32_O_16_	3.10	1.59	Raffinose	[49]
B3	1.88_404.1312n	−1.68	C_17_H_24_O_11_	1.88	1.64	Gardenoside	*
B4	3.44_406.1255n	−2.11	C_20_H_22_O_9_	3.75	2.29	Astringin	[50]
B5	3.87_390.1309n	−1.34	C_20_H_22_O_8_	1.06	3.54	Piceid	[51]
B6	4.28_244.0731n	−2.06	C_14_H_12_O_4_	4.38	2.05	Oxyresveratrol	#
B7	5.27_458.1572n	−1.03	C_24_H_26_O_9_	2.04	2.24	Mulberroside C	*
B8	6.12_325.1078*m*/*z*	−1.2	C_19_H_18_O_5_	1.33	4.27	Moracinfurol A	*

n neutral molecular weight calculated according to adduct ion forms; # confirmed with the MS and MS/MS spectra of reference standard; * confirmed with the mass error (ME), fragmentation score (FS), isotopic similarity (IS), and score of matched with an in-house library.

## Data Availability

The data presented in this study are available on request from the corresponding author. The data are not publicly available due to privacy.

## Supplementary Materials

The following supporting information can be downloaded at: https://www.mdpi.com/article/10.3390/metabo12020106/s1, Figure S1: TIC of metabolites in leave and stem bark samples (take LF21 and BF21 for example): (A) the whole TIC of LF21; (B) part of TIC at RT 2.0–5.0 min of LF21; (C) the whole TIC of BF21, Figure S2: Permutation test with 200 permutations: (A) permutation test of OPLS-DA model for leaves; (B) permutation test of OPLS-DA model for stem barks, Table S1: Identified metabolites in leaf samples, Table S2: Identified metabolites in stem bark samples, Table S3: Semi-quantitation of oxyresveratrol in stem bark samples (μg/g), Table S4: Antioxidant activity of male and female leaf samples (mg/g), Table S5: Antioxidant activity of male and female stem bark samples (mg/g), Table S6: Student’ *t*-test of antioxidant activity of male and female leaf samples, Table S7: Student’ t-test of antioxidant activity of male and female stem bark samples.
