# Supplementary material for: Untargeted Metabolomics Coupled with Chemometrics for Leaves and Stem Barks of Dioecious Morus alba L
Source: Metabolites. 2022 Jan 24;12(2):106. doi: 10.3390/metabo12020106 (PMC8874686; doi:10.3390/metabo12020106)
Supplement: Supplementary file 1 [file metabolites-12-00106-s001.zip › metabolites-1537928-supplementary.pdf]

Untargeted Metabolomics Coupled with Chemometrics for Leaves and Stem Barks of Dioecious *Morus alba* L.

Cui Wu, Huijun Wang, Zhenying Liu, Bo Xu, Zhuojun Li, Pingping Song, and Zhimao Chao*

Institute of Chinese Materia Medica, China Academy of Chinese Medical Sciences, Beijing 100700, China; wucuidalian@163.com; hjwang0819@icmm.ac.cn; liuzy9607@163.com; xubo_345@163.com; 18811385399@163.com; songpingping122@163.com; zmchao@icmm.ac.cn

* Correspondence: zmchao@icmm.ac.cn (Z.C.)

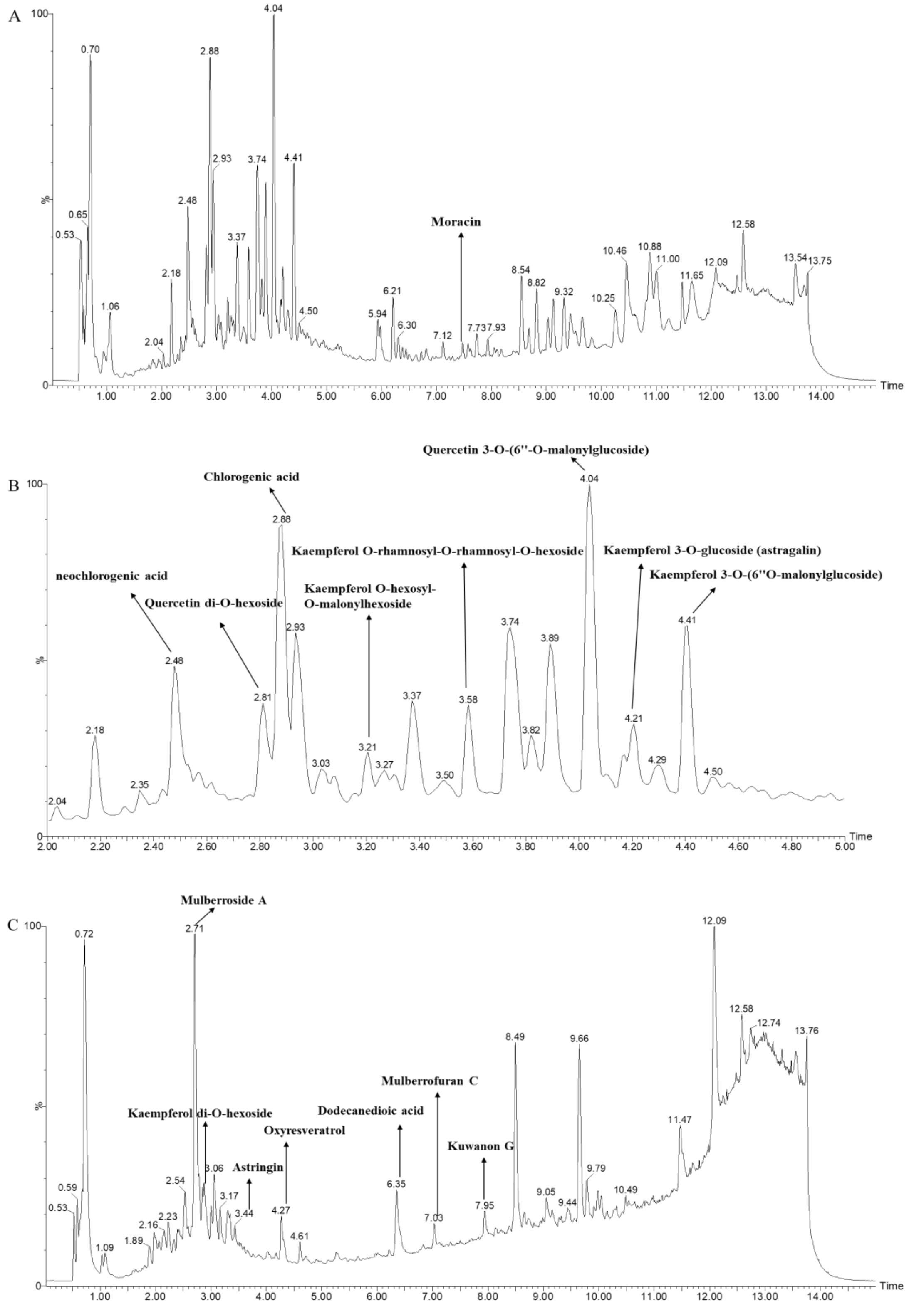

Figure S1. TIC of metabolites in leaf and stem bark samples (take LF21 and BF21 for example): (A) the whole TIC of LF21; (B) TIC at retention time of 2.0-5.0 min of LF21; (C) the whole TIC of BF21

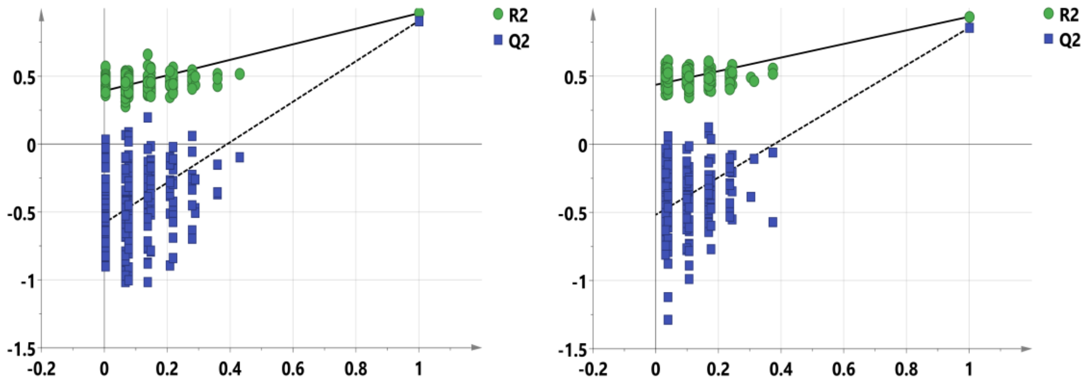

(A) (B)

Figure S2. Permutation test with 200 permutations: (A) permutation test of OPLS-DA model for leaves; (B) permutation test of OPLS-DA model for stem barks

Table **S1** Identified metabolites in leaf samples

No.	RT [a] (min)	*m/z* or n (Da)	Theoretical exact mass (Da)	Mass accuracy (ppm)	Formula	Compound	Class
1	0.61	180.0630	180.0647	−9.39	$C_7H_8N_4O_2$	Theophylline[3]	Alkaloids
2	0.62	132.0300	132.0297	−1.35	$C_4H_7NO_4$	Aspartic acid[2]	Amino acid
3	0.63	275.0534	275.0556	−9.98	$C_7H_6O_3$	Protocatechuic aldehyde[3]	Aldehydes
4	0.64	195.0507	195.0505	−1.47	$C_6H_{12}O_7$	Gulonic acid[2]	Organic acids
5	0.70	191.0555	191.0556	−3.13	$C_7H_{12}O_6$	Quinic acid[2]	Phenolic acids
6	0.71	504.1687	504.1690	−0.67	$C_{18}H_{32}O_{16}$	Raffinose[2]	Saccharides
7	0.72	179.0558	179.0556	−1.94	$C_3H_6O_3$	Hydroxypropionic acid[3]	Organic acids
8	0.72	209.0663	209.0661	−2.25	$C_6H_{12}O_5$	Rhamnose[2]	Saccharides
9	0.74	173.0089	173.0086	−1.49	$C_6H_8O_7$	Citric acid[2]	Organic acids
10	0.75	134.0209	134.0215	−4.43	$C_4H_6O_5$	Malic acid[2]	Organic acids
11	1.06	111.0082	111.0082	−4.85	$C_5H_4O_3$	Furoic acid[2]	Organic acids
12	1.62	312.0945	312.0944	−1.73	$C_{10}H_{13}N_5O_4$	Adenosine[2]	Alkaloids
13	1.87	331.1030	331.1029	−1.71	$C_{13}H_{18}O_7$	Salicin[2]	Benzylalcohol glucosides
14	1.88	139.0399	139.0395	−1.29	$C_7H_{10}O_4$	Terebic Acid[3]	Organic acids
15	1.93	153.0190	153.0188	−2.17	$C_7H_6O_4$	Protocatechuic acid[2]	Phenolic acids
16	2.18	359.0979	359.0978	−1.38	$C_{14}H_{18}O_8$	Pungenin[3]	Acetophenone glucosides
17	2.48	353.0876	353.0873	−0.62	$C_{16}H_{18}O_9$	Neochlorogenic acid[2]	Phenolic acids
18	2.57	203.0824	203.0821	−0.85	$C_{11}H_{12}N_2O_2$	Tryptophan[2]	Amino acids
19	2.57	918.2618	918.2641	−2.57	$C_{39}H_{50}O_{25}$	Quercetin di O-rhamnosyl-O-hexoside[2]	Flavonol glycosides
20	2.60	471.1143	471.1139	−0.15	$C_{20}H_{24}O_{13}$	5,7-Dihydroxycoumarin 7-(6-O-*β*-D-apiofuranosyl-*β*-D-glucopyranoside)[3]	Coumarins
21	2.64	679.1857	679.1816	5.28	$C_{19}H_{16}O_6$	Dehydrocyclomorusin[3]	Flavones
22	2.64	737.1904	737.1929	−4.06	$C_{33}H_{40}O_{20}$	Quercetin O-rhamnosyl-O-rhamnosyl-O-hexoside[2]	Flavonol glycosides
23	2.67	568.1787	568.1792	−0.97	$C_{26}H_{32}O_{14}$	Mulberroside A[1]	Stilbenes
24	2.70	901.2588	901.2614	−3.50	$C_{39}H_{50}O_{24}$	Kaempferol di O-rhamnosyl-O-hexoside[2]	Flavonol glycosides
25	2.71	755.2030	755.2035	−1.39	$C_{33}H_{40}O_{20}$	Kaempferol O-rhamnosyl-O-hexosyl-O-hexoside[2]	Flavonol glycosides
26	2.75	857.1984	857.1988	−1.08	$C_{36}H_{42}O_{24}$	Kaempferol O-hexosyl-O-hexosyl-O-malonylhexoside[2]	Flavonol glycosides
27	2.77	161.0244	161.0239	−0.25	$C_9H_6O_3$	Umbelliferone[2]	Coumarins
28	2.77	369.0826	369.0822	−0.33	$C_{15}H_{16}O_8$	Skimmin[2]	Coumarins
29	2.78	137.0241	137.0239	−2.24	$C_7H_6O_3$	*p*-Hydroxybenzoic acid[2]	Phenolic acids
30	2.78	595.1307	595.1299	0.38	$C_{26}H_{28}O_{16}$	Luteolin-7-diglucoside[2]	Flavone glycosides

31	2.80	772.2058	772.2062	−0.50	$C_{33}H_{40}O_{21}$	Quercetin O-rhamnosyl-O-hexosyl-O-hexoside-I[2]	Flavonol glycosides
32	2.81	626.1483	626.1483	−0.05	$C_{27}H_{30}O_{17}$	Quercetin di-O-hexoside[2]	Flavonol glycosides
33	2.85	465.1037	465.1033	−0.38	$C_{21}H_{22}O_{12}$	Plantagoside[3]	Flavanone glycosides
34	2.87	161.0241	161.0239	−1.70	$C_9H_8O_4$	Umbellic acid[3]	Phenolic acids
35	2.87	707.1825	707.1823	−0.59	$C_{16}H_{18}O_9$	Chlorogenic acid[1]	Phenolic acids
36	2.91	359.0772	359.0767	−0.01	$C_{18}H_{16}O_8$	Rosmarinic acid[2]	Phenolic acids
37	2.92	289.0722	289.0712	1.79	$C_{14}H_{12}O_4$	Piceatannol[2]	Stilbenes
38	2.94	163.0398	163.0395	−1.83	$C_9H_8O_3$	*p*-Coumaric acid[2]	Phenolic acids
39	2.94	529.1559	529.1557	−0.85	$C_{22}H_{28}O_{12}$	6,7-Dihydroxycoumarin 7-(6-O-α-rhamnopyranosyl-β-D-glucopyranoside)[3]	Coumarins
40	2.96	711.1413	711.1409	−0.13	$C_{30}H_{32}O_{20}$	Quercetin O-hexosyl-O-malonylhexoside[2]	Flavonol glycosides
41	3.04	566.1602	566.1636	−5.84	$C_{26}H_{30}O_{14}$	Mulberroside F[3]	Benzofurans
42	3.20	353.0876	353.0873	−0.64	$C_{16}H_{18}O_9$	Cryptochlorogenic acid2	Phenolic acids
43	3.20	771.1982	771.1984	−0.97	$C_{33}H_{40}O_{21}$	Quercetin O-rhamnosyl-O-hexosyl-O-hexoside- II [2]	Flavonol glycosides
44	3.21	695.1492	695.1460	3.93	$C_{30}H_{32}O_{19}$	Kaempferol O-hexosyl-O-malonylhexoside[2]	Flavonol glycosides
45	3.36	386.1936	386.1941	−1.13	$C_{19}H_{30}O_8$	Roseoside[2]	Sesquiterpenoids
46	3.45	227.0715	227.0708	0.46	$C_{14}H_{12}O_3$	Resveratrol[2]	Stilbenes
47	3.50	463.0882	463.0877	0.04	$C_{21}H_{20}O_{12}$	Hyperoside[2]	Flavonol glycosides
48	3.58	739.2090	739.2086	−0.07	$C_{33}H_{40}O_{19}$	Kaempferol O-rhamnosyl-O-rhamnosyl-O-hexoside[2]	Flavonol glycosides
49	3.63	679.2597	679.2543	7.16	$C_{20}H_{20}O_5$	Euchrenone a7[3]	Flavanones
50	3.77	695.1473	695.1460	1.18	$C_{30}H_{32}O_{19}$	Quercetin O-rhamnosyl-O-malonylhexoside[2]	Flavonol glycosides
51	3.82	593.1516	593.1506	0.70	$C_{27}H_{30}O_{15}$	Kaempferol O-rhamnosyl-O-hexoside[2]	Flavonol glycosides
52	4.03	550.0963	550.0959	0.83	$C_{24}H_{22}O_{15}$	Quercetin 3-O-(6''-O-malonylglucoside)[2]	Flavonol glycosides
53	4.03	679.1530	679.1510	2.10	$C_{30}H_{32}O_{18}$	Kaempferol O-rhamnosyl-O-malonylhexoside[2]	Flavonol glycosides
54	4.12	303.0511	303.0505	0.32	$C_{15}H_{12}O_7$	Taxifolin[2]	Flavanones
55	4.21	448.1007	448.1006	0.25	$C_{21}H_{20}O_{11}$	Kaempferol 3-O-glucoside (astragalin)[2]	Flavonol glycosides
56	4.22	243.0665	243.0657	0.85	$C_{14}H_{12}O_4$	Oxyresveratrol[1]	Stilbenes
57	4.29	417.1581	417.1549	6.21	$C_{22}H_{26}O_8$	Syringaresinol[3]	Lignans
58	4.30	507.1056	507.1080	−5.83	$C_{15}H_{10}O_4$	Chrysin[2]	Flavones

59	4.40	534.1013	534.1010	0.72	$C_{24}H_{22}O_{14}$	Kaempferol 3-O-(6''-O-malonylglucoside)[2]	Flavonol glycosides
60	5.06	241.0507	241.0501	0.47	$C_{14}H_{10}O_4$	5-(5-Hydroxybenzofuran-2-yl) benzen-1,3-diol[3]	Benzofurans
61	5.20	161.0244	161.0239	−0.38	$C_9H_6O_3$	7-Hydroxycoumarin[3]	Coumarins
62	5.24	285.0407	285.0399	0.78	$C_{15}H_{10}O_6$	Luteolin[1]	Flavones
63	5.60	271.0613	271.0606	0.47	$C_{15}H_{12}O_5$	Naringenin[1]	Flavones
64	5.83	269.0454	269.0450	−0.44	$C_{15}H_{10}O_5$	Apigenin[1]	Flavones
65	7.47	309.1130	309.1127	−0.59	$C_{19}H_{18}O_4$	Moracin[3]	Benzofurans
66	8.20	311.2214	311.2222	−4.33	C_9H16O_2	6-Hydroxy-4,6-dimethyl-3-hepten-2-one[3]	Ketones

a RT Retention time; 1 Confirmed with the MS and MS/MS spectra with reference standards; 2 Confirmed with the MS data with literatures; 3 Confirmed with the mass error (ME), fragmentation score (FS), isotopic similarity (IS), retention time (RT), score of matched with in-house library or wed-accessible database.

Table S2 Identified metabolites in stem bark samples

No.	RT [a] (min)	*m/z* or n (Da)	Theoretical exact mass (Da)	Mass accuracy (ppm)	Formula	Compound	Class
1	0.60	173.1038	173.1039	−3.47	$C_6H_{14}N_4O_2$	Arginine[2]	Amino acids
2	0.64	259.0220	259.0219	−1.64	$C_6H_{13}O_9P$	*β*-D-Glucose 1-phosphate[3]	Organic acids
3	0.65	146.0454	146.0453	−3.36	$C_5H_9NO_4$	Glutamic acid[2]	Amino acids
4	0.66	421.0749	421.0771	−6.51	$C_{19}H_{18}O_{11}$	Mangiferin[2]	Xanthone glycosides
5	0.67	195.0504	195.0505	−3.13	$C_6H_{12}O_7$	Gulonic acid[2]	Organic acids
6	0.67	209.0297	209.0297	−2.76	$C_6H_{10}O_8$	Mucic acid[3]	Organic acids
7	0.68	165.0399	165.0399	−3.42	$C_5H_{10}O_6$	Xylonic acid[3]	Organic acids
8	0.69	135.0293	135.0293	−4.25	$C_4H_8O_5$	Threonic acid[2]	Organic acids
9	0.72	191.0554	191.0556	−3.66	$C_7H_{12}O_6$	Quinic acid[2]	Phenolic acids
10	0.75	504.1689	504.1690	−0.23	$C_{18}H_{32}O_{16}$	Raffinose[2]	Saccharides
11	0.76	134.0212	134.0215	−2.83	$C_4H_6O_5$	Malic Acid[2]	Organic acids
12	1.10	192.0266	192.0270	−2.00	$C_6H_8O_7$	Isocitric acid[2]	Organic acids
13	1.46	243.0619	243.0617	−1.61	$C_9H_{12}N_2O_6$	Uridine[2]	Alkaloids
14	1.65	134.0467	134.0467	−3.60	$C_5H_5N_5$	Adenine[2]	Alkaloids
15	1.65	312.0944	312.0944	−2.10	$C_{10}H_{13}N_5O_4$	Adenosine[2]	Alkaloids
16	1.70	282.0840	282.0838	−1.25	$C_{10}H_{13}N_5O_5$	Guanosine[2]	Alkaloids
17	1.88	404.1312	404.1319	−1.68	$C_{17}H_{24}O_{11}$	Gardenoside[2]	Iridoids
18	1.93	419.1195	419.1190	−0.14	$C_{16}H_{22}O_{10}$	Gardoside[3]	Iridoids
19	2.21	339.0731	339.0716	2.65	$C_{15}H_{16}O_9$	Aesculin[2]	Coumarins
20	2.61	449.1084	449.1084	−1.12	$C_{21}H_{22}O_{11}$	Astilbin[2]	Flavanonol glycosides
21	2.71	1135.3450	2M-H	−5.43	$C_{26}H_{32}O_{14}$	Mulberroside A[1]	Stilbenes
22	2.89	465.1031	465.1033	−1.56	$C_{21}H_{22}O_{12}$	Plantagoside[3]	Flavanone glycosides
23	2.89	625.1405	625.1405	−0.81	$C_{27}H_{30}O_{17}$	Kaempferol di-O-hexoside[2]	Flavonol glycosides
24	2.91	354.0945	354.0951	−1.53	$C_{16}H_{18}O_9$	Chlorogenic acid[1]	Phenolic acids
25	3.09	566.1651	566.1636	2.66	$C_{26}H_{30}O_{14}$	Mulberroside F[3]	Benzofurans
26	3.32	449.1084	449.1084	−1.41	$C_{20}H_{20}O_9$	Moracin M 3'-O-*β*-glucopyranoside[3]	Benzofurans
27	3.38	433.1136	433.1135	−0.95	$C_{21}H_{22}O_{10}$	Prunin[2]	Flavanone glycosides
28	3.44	406.1255	406.1264	−2.11	$C_{20}H_{22}O_9$	Astringin[2]	Stilbenes
29	3.47	273.0763	273.0763	−2.24	$C_{14}H_{12}O_3$	Resveratrol[2]	Stilbenes
30	3.50	435.1284	435.1291	−2.87	$C_{21}H_{24}O_{10}$	Phloridzin[2]	Dihydrochalcone glycosides
31	3.54	463.0876	463.0877	−1.27	$C_{21}H_{20}O_{12}$	Hyperoside[2]	Flavonol glycosides
32	3.87	390.1309	390.1315	−1.34	$C_{20}H_{22}O_8$	Piceid[2]	Stilbenes
33	4.07	403.1030	403.1029	−1.18	$C_{20}H_{22}O_{10}$	Oxyresveratrol 3'-O-*β*-D-glucoside[3]	Stilbenes
34	4.28	244.0731	244.0736	−2.06	$C_{14}H_{12}O_4$	Oxyresveratrol[1]	Stilbenes
35	5.27	241.1077	241.1076	−2.14	$C_{11}H_{16}O_3$	Loliolide[2]	Monoterpenoids
36	5.27	325.1077	325.1076	−1.35	$C_{19}H_{18}O_5$	Wittifuran V[3]	Benzofurans

37	5.27	458.1572	458.1577	−1.03	$C_{24}H_{26}O_9$	Mulberroside C[3]	Benzofurans
38	6.12	325.1078	325.1076	−1.20	$C_{19}H_{18}O_5$	Moracinfurol A[3]	Benzofurans
39	7.03	579.1655	579.1655	−0.90	$C_{34}H_{28}O_9$	Mulberrofuran C[3]	Benzofurans
40	6.36	230.1513	230.1518	−2.29	$C_{12}H_{22}O_4$	Dodecanedioic acid[3]	Organic acids
41	7.95	691.2178	691.2179	−1.05	$C_{40}H_{36}O_{11}$	Kuwanon G[1]	Flavones
42	8.02	351.0869	351.0869	−1.55	$C_{20}H_{16}O_6$	Cyclocommunol[3]	Flavones
43	8.11	435.1441	435.1444	−1.83	$C_{25}H_{24}O_7$	10-Oxomornigrol F[3]	Flavones
44	8.14	437.1597	437.1600	−2.02	$C_{25}H_{26}O_7$	Mornigrol F[3]	Flavones

a RT Retention time; 1 Confirmed with the MS and MS/MS spectra with reference standards; 2 Confirmed with the MS data with literatures; 3 Confirmed with the mass error (ME), fragmentation score (FS), isotopic similarity (IS), retention time (RT), score of matched with in-house library or wed-accessible database.

Table S3 Semi-quantitation of oxyresveratrol in stem bark samples (μg/g)

No.	Content [a]	No.	Content [a]	No.	Content [a]	No.	Content [a]	No.	Content [a]
BF1	11.23	BF14	4.522	BF27	6.02	BM3	3.29	BM16	2.39
BF2	9.43	BF15	12.14	BF28	17.66	BM4	5.76	BM17	3.25
BF3	6.12	BF16	19.00	BF29	2.08	BM5	1.04	BM18	4.30
BF4	15.99	BF17	19.73	BF30	5.44	BM6	1.62	BM19	0.77
BF5	13.41	BF18	8.27	BF31	5.51	BM7	1.30	BM20	3.89
BF6	7.18	BF19	5.96	BF32	8.36	BM8	2.90	BM21	1.71
BF7	12.11	BF20	6.33	BF33	13.48	BM9	5.41	BM22	3.82
BF8	15.56	BF21	6.64	BF34	5.17	BM10	0.86	BM23	7.68
BF9	7.90	BF22	15.45	BF35	10.93	BM11	1.07	BM24	4.32
BF10	24.18	BF23	10.61	BF36	18.12	BM12	1.96		
BF11	4.77	BF24	22.27	BF37	13.63	BM13	2.01		
BF12	19.20	BF25	11.25	BM1	1.33	BM14	3.21		
BF13	15.76	BF26	6.09	BM2	0.87	BM15	8.48		

a Contents was calculated by comparing the peak areas of standard and normalized peak areas of samples

Table S4 Antioxidant activity values of male and female leaf samples (mg/g)

No.	DPPH [a]	ABTS [a]	No.	DPPH [a]	ABTS [a]	No.	DPPH [a]	ABTS [a]
LF1	24.72±0.41	18.62±0.70	LF21	10.90±0.49	11.16±0.68	LM7	20.94±0.38	19.24±0.62
LF2	34.78±0.51	21.26±0.42	LF22	24.70±0.49	21.83±0.43	LM8	7.40±0.48	10.07±0.33
LF3	22.64±0.51	19.83±0.46	LF23	13.79±0.49	16.79±0.3	LM9	14.13±0.27	15.04±0.74
LF4	28.44±0.41	23.33±0.70	LF24	11.85±0.49	18.84±0.6	LM10	11.28±0.37	15.24±0.21
LF5	14.81±0.51	15.84±0.55	LF25	20.56±0.3	21.00±0.51	LM11	15.38±0.37	13.73±0.37
LF6	15.30±0.41	16.70±0.34	LF26	20.63±0.4	14.97±0.43	LM12	9.63±0.48	11.65±0.21
LF7	20.15±0.41	18.66±0.34	LF27	17.39±0.3	16.95±0.76	LM13	18.76±0.37	16.78±0.5
LF8	14.81±0.51	13.16±0.30	LF28	22.23±0.3	19.85±0.64	LM14	16.45±0.43	17.46±0.54
LF9	16.86±0.41	15.83±0.22	LF29	16.48±0.4	16.85±0.47	LM15	13.17±0.43	17.55±0.25
LF10	15.90±0.41	17.39±0.34	LF30	24.65±0.4	19.49±0.47	LM16	12.39±0.37	12.68±0.37
LF11	8.88±0.21	16.61±0.22	LF31	27.23±0.3	18.55±0.55	LM17	17.84±0.37	15.43±0.25
LF12	6.45±0.41	14.37±0.76	LF32	18.86±0.4	18.47±0.26	LM18	18.72±0.43	17.35±0.33
LF13	7.16±0.41	15.29±0.72	LF33	19.81±0.4	19.22±0.26	LM19	16.98±0.43	16.55±0.54
LF14	10.09±0.51	16.22±0.60	LF34	14.56±0.4	15.34±0.68	LM20	18.06±0.27	17.88±0.45
LF15	17.52±0.51	12.08±0.72	LM1	20.19±0.43	16.03±0.50	LM21	16.21±0.32	16.39±0.49
LF16	24.81±0.40	18.40±0.43	LM2	16.33±0.47	15.89±0.45	LM22	10.64±0.32	13.21±0.54
LF17	15.36±0.40	15.05±0.30	LM3	13.12±0.43	11.92±0.41	LM23	15.36±0.43	13.45±0.45
LF18	23.25±0.40	20.61±0.38	LM4	13.45±0.39	14.55±0.37	LM24	14.90±0.38	15.77±0.41
LF19	23.17±0.49	23.08±0.51	LM5	18.47±0.27	17.50±0.37			
LF20	18.65±0.49	19.78±0.60	LM6	19.17±0.32	17.09±0.37			

a Data are represented as the mean ± SD (*n*=3)

Table S5 Antioxidant activity values of male and female stem bark samples (mg/g)

No.	DPPH [a]	ABTS [a]	No.	DPPH [a]	ABTS [a]	No.	DPPH [a]	ABTS [a]
BF1	1.27±0.09	7.58±0.37	BF22	1.30±0.11	16.48±0.34	BM6	1.39±0.09	10.30±0.21
BF2	1.41±0.11	8.04±0.37	BF23	0.82±0.09	13.35±0.38	BM7	0.87±0.10	10.42±0.46
BF3	1.13±0.09	6.27±0.33	BF24	1.84±0.11	15.27±0.26	BM8	1.55±0.10	12.39±0.21
BF4	1.38±0.10	7.32±0.41	BF25	1.24±0.12	14.34±0.47	BM9	1.18±0.12	8.86±0.41
BF5	1.60±0.08	8.52±0.25	BF26	1.66±0.06	15.67±0.38	BM10	1.20±0.11	8.37±0.41
BF6	0.88±0.11	14.06±0.47	BF27	1.26±0.09	13.18±0.42	BM11	0.77±0.09	9.01±0.45
BF7	0.75±0.09	13.41±0.22	BF28	1.03±0.11	11.57±0.38	BM12	1.47±0.09	13.06±0.33
BF8	1.22±0.08	14.11±0.34	BF29	2.18±0.11	16.36±0.21	BM13	1.28±0.10	11.78±0.54
BF9	1.31±0.11	15.21±0.26	BF30	3.25±0.09	21.99±0.34	BM14	1.34±0.14	11.49±0.50
BF10	1.04±0.10	14.37±0.26	BF31	1.50±0.09	14.68±0.43	BM15	1.50±0.12	11.93±0.25
BF11	1.18±0.10	15.51±0.3	BF32	1.03±0.11	11.23±0.26	BM16	1.60±0.11	10.00±0.41
BF12	1.02±0.11	13.95±0.34	BF33	2.31±0.11	16.68±0.3	BM17	0.88±0.09	12.22±0.5
BF13	1.54±0.11	15.28±0.34	BF34	3.68±0.09	16.59±0.38	BM18	0.89±0.13	11.16±0.37
BF14	1.23±0.11	12.66±0.34	BF35	2.50±0.15	21.08±0.26	BM19	0.78±0.09	11.43±0.25
BF15	0.99±0.11	14.56±0.34	BF36	1.42±0.11	22.00±0.34	BM20	1.40±0.11	10.25±0.21
BF16	0.94±0.08	13.90±0.38	BF37	1.82±0.11	26.11±0.38	BM21	1.18±0.11	13.47±0.5
BF17	2.24±0.10	19.19±0.43	BM1	0.92±0.12	9.22±0.41	BM22	1.55±0.12	13.99±0.41
BF18	1.72±0.09	18.03±0.47	BM2	1.00±0.11	8.06±0.25	BM23	1.39±0.11	12.58±0.41
BF19	1.52±0.10	15.30±0.43	BM3	1.45±0.12	8.17±0.33	BM24	1.05±0.10	14.25±0.41
BF20	1.23±0.07	15.72±0.34	BM4	1.22±0.12	9.13±0.54			
BF21	1.68±0.07	17.71±0.38	BM5	0.91±0.1	9.67±0.25			

a Data are represented as the mean ± SD (n=3)

Table S6 Student' t-test of antioxidant activity of male and female leaf samples

Methods	Mean ± SD Male (*n*=24)	Mean ± SD Female (*n*=34)	*t*	*p*
DPPH	15.37±3.49	18.45±6.34	-2.371	0.021
ABTS	15.35±2.29	17.69±2.91	-3.283	0.002

Table S7 Student' t-test of antioxidant activity of male and female stem bark samples

Methods	Mean ± SD Male (*n*=24)	Mean ± SD Female (*n*=37)	*t*	*p*
DPPH	1.20±0.27	1.52±0.64	-2.714	0.009
ABTS	10.88±1.87	14.79±4.18	-4.977	0.000
